# The utility of the “consideRATE” patient-reported experience measure in hospital patients receiving specialist palliative care: a two-phase prospective pilot study

**DOI:** 10.3389/frhs.2026.1933654

**Published:** 2026-09-30

**Authors:** Claudia Virdun, Davinia Seah, Mona Ramsay, Richard Chye, Annmarie Hosie

**Affiliations:** 1 Flinders Research Centre for Palliative Care, Death, and Dying, College of Health and Enablement, Flinders University, Adelaide, SA, Australia; 2 Faculty of Health, Cancer and Palliative Care Outcomes Centre, Queensland University of Technology (QUT), Brisbane, QLD, Australia; 3 Sacred Heart Supportive and Palliative Care, St. Vincent's Hospital Sydney (SVHS), Darlinghurst, NSW, Australia; 4 School of Nursing & Midwifery, University of Notre Dame Australia, Sydney, NSW, Australia; 5 IMPACCT (Improving Palliative, Aged and Chronic Care through Clinical Research and Translation), Faculty of Health, University of Technology Sydney, Sydney, NSW, Australia

**Keywords:** hospitals, palliative care, patient-centered care, quality improvement, quality of health care

## Abstract

**Background:**

Patient-reported experience measures (PREMs) are increasingly central to ensuring healthcare quality. Uncertainty about the utility of PREMs for hospital patients with palliative care needs has limited their use in this population.

**Aim:**

To determine the utility (feasibility, acceptability, and appropriateness) of implementing the PREM “consideRATE” for patients receiving specialist palliative care and to explore its perceived value for quality improvement.

**Design:**

A two-phase prospective pilot study incorporating PREM administration, screening logs and field notes, and focus groups.

**Setting/participants:**

The study was conducted in a specialist palliative care unit and consultative service in a metropolitan Australian hospital. Participants were recruited by research staff and included adult inpatients receiving specialist palliative care or their carers (PREM completion and feedback) and multidisciplinary staff (clinicians, managers, and quality and governance personnel) (focus groups).

**Results:**

Across 219 eligible participation occasions, 188 PREMs were completed (86% participation rate). No patient or carer distress was reported during PREM administration, and occasional spontaneous comments recorded in field notes indicated appreciation for the opportunity to provide feedback. Staff participants viewed consideRATE as meaningful and actionable, and perceived that the data could support the identification of improvement priorities and advocacy for system change. They also stated that administration support was essential and that routine implementation of consideRATE was not feasible within existing clinical workloads.

**Conclusions:**

Supported administration of consideRATE in a specialist palliative care unit and consultative service resulted in high participation and completion rates with no reported distress among participating patients and carers. Staff considered the measure appropriate and perceived the data as useful for informing quality improvement, but concluded that sustainable routine implementation would require dedicated human resources and integration into the hospital's quality systems.

## Introduction

Measuring patient experience has become a central component of healthcare quality appraisal internationally, reflecting a shift toward person-centered, values-based health systems (1, 2). Patient-reported experience measures (PREMs) are now widely embedded in hospital performance frameworks, accreditation standards, and quality improvement systems, with the intent of capturing how care is perceived and experienced by those receiving it (3, 4). Increasingly, PREMs are recognized as essential complements to clinical outcomes and safety indicators, particularly in complex care contexts where technical measures alone are insufficient to capture what matters most to patients and families (5, 6).

Despite this policy momentum, patients with palliative care needs have remained largely excluded from routine patient experience measurement in acute hospital settings (7–11). This exclusion persists even though people with palliative care needs represent a substantial proportion of hospital inpatients and often experience prolonged, repeated, and highly complex interactions with health services (12–16). In many institutions, standard PREMs are administered shortly after discharge, rely on digital or self-completion formats, and are designed for general inpatient populations (5, 6, 17, 18). As a result, they are often inaccessible to patients who are seriously ill, fatigued, cognitively impaired, or approaching the end of life, as well as for those who die during admission (8, 9). Exclusion of patients with palliative care needs from PREMs is commonly driven by uncertainty about the feasibility, acceptability, appropriateness, and organizational readiness of this feedback process.

The absence of systematic experience data in palliative care has important implications for healthcare quality and safety (8, 9, 19). Without input from patients with palliative care needs, health services lack meaningful insight into the quality of care delivered at a time when communication, symptom control, respectful and compassionate care, emotional support, and preparation for deterioration are particularly important (19–23). Studies consistently demonstrate that hospital care for people with palliative care needs is often characterized by unmet needs, fragmented communication, variable symptom management, and limited attention to psychosocial and existential concerns (20, 24, 25). This variation in care experience is related to the complexity of care needs for those with advanced illness cared for within busy systems designed for acute and episodic care (26).

Palliative care-specific PREMs are emerging in response to the growing recognition of this gap (10). These tools seek to capture domains of care that are central to the palliative experience but poorly assessed by existing generic instruments (10). However, questions remain regarding which tools are most appropriate, how they can be integrated into routine organizational systems, and whether they are feasible and acceptable within real-world acute care contexts (8–10, 27). Members of the current research team have worked to establish the feasibility and acceptability of one PREM designed for people living with advanced illness, consideRATE (28), within one large hospital setting (8, 9). This work suggests that consideRATE can generate meaningful experience data for people living with advanced illness, is valued by both patients and clinicians, and can inform quality improvement efforts (8, 19). However, uncertainties remain regarding consideRATE's utility in other hospital contexts, including how such data can be collected and embedded in existing quality and reporting systems (8). Addressing these questions is a necessary precursor to broader implementation efforts. This two-phase prospective pilot study responded to this need by evaluating the feasibility and acceptability of supported administration of consideRATE for patients receiving specialist palliative care in the hospital and exploring staff perspectives regarding its appropriateness, implementation, and perceived usefulness for informing quality improvement.

## Methods

This was a two-phase prospective pilot study (29) incorporating consideRATE administration, screening log and field note completion, and focus groups. Phase 1 was conducted within the inpatient specialist palliative care unit to test recruitment processes among patients identified as ready for discharge. Findings from Phase 1 informed revisions to eligibility criteria and recruitment procedures. Phase 2 consisted of three components: (a) PREM collection within the inpatient palliative care unit using revised eligibility criteria; (b) PREM collection among patients receiving specialist palliative care via the hospital consultative service; and (c) focus groups with clinicians, managers, and quality personnel to explore implementation experiences and the perceived value of PREM data for quality improvement.

### Setting

The study was conducted in a specialist 13-bed inpatient palliative care unit and a consultative service in a 460-bed tertiary referral hospital in Sydney, Australia. The inpatient unit is located across the road from the tertiary hospital, where the on-call 24/7 consultative specialist palliative care team has admission rights. Of note, patients receiving consultative palliative care in the tertiary hospital typically receive specialist medical and nursing review but lack access to the full interdisciplinary palliative care team, and their day-to-day care is delivered by other clinical staff.

### Participants

*PREM completion:* Inpatients receiving specialist palliative care were eligible to complete consideRATE if they were aged ≥18 years or an adult carer and able to provide informed consent. Eligibility changed across study phases in relation to patients/carers (Table 1).

**Table 1:** Eligibility for PREM completion.

Phase 1 (3 months)	Phase 2 (6 months)
Patients/carers Adult inpatients on the specialist palliative care unit Noted by the medical team to be ready for discharge within the next 48 h If the inpatient returned to the unit within Phase 1, they were eligible to complete the PREM again If the patient was unable to complete the PREM due to impaired cognition, fatigue or distress, their carer was able to complete it by proxy	Patients/carers Adult inpatients on the specialist palliative care unit (Phase 2a) Adult inpatients of the tertiary hospital referred to the specialist palliative care consultative service (Phase 2b) Relevant to phases 2a and 2b: An inpatient for ≥48 h (regardless of plans for discharge) If an extended stay, the inpatient was able to repeat PREM completion (not more than once per week) If the patient was unable to complete the PREM due to impaired cognition, fatigue, or distress, their carer was able to complete it by proxy

*Focus groups (Phase 2c):* Multidisciplinary clinicians, clinical managers, and quality and governance personnel (“staff) supporting the care of patients with palliative care needs and able to influence improvement work.

### Sample size and sampling

As a two-phase prospective pilot study, formal power calculations were not undertaken. Phase 1 used total sampling of all eligible discharges over a 3-month period and was anticipated to yield approximately 36 participants. Phase 2 aimed to recruit 80 patients and 10 staff participants, with these samples expected to provide sufficient data to descriptively assess study outcomes.

### Implementation outcomes and operationalization

This study was informed by Proctor et al.'s (30) framework, which distinguishes implementation outcomes from clinical and service outcomes. Three implementation outcomes were examined: feasibility, acceptability, and appropriateness. When considered collectively, these outcomes inform the “utility” of a process being implemented, especially when the perspectives of recipients, providers, and organizational decision makers are all captured (31). In addition, an exploratory outcome relating to the perceived usefulness of consideRATE data for quality improvement was examined through staff focus groups.

*Feasibility* was defined as the extent to which supported administration of consideRATE could be successfully undertaken for patients receiving specialist palliative care and the factors perceived by staff to influence future routine implementation. Participation was defined as the proportion of eligible participation occasions that agreed to complete consideRATE. Completion was defined as the proportion of consenting participation occasions that completed all consideRATE questions. Predefined feasibility thresholds were >70% participation among eligible occasions and >80% full completion among consenting occasions.

*Acceptability* was defined as the extent to which participants were willing to engage with consideRATE and their perceptions of the administration process. In this study, acceptability was assessed through participation and completion rates, the absence of observed or reported distress during PREM administration, spontaneous participant feedback documented in field notes, and staff participants' perspectives on its relevance and acceptability.

*Appropriateness* was defined as the perceived fitness, relevance, and usefulness of consideRATE for patients receiving specialist palliative care within the hospital context. Here, appropriateness was explored primarily through staff perspectives, including the perceived relevance of consideRATE's content and its usefulness for informing care delivery and quality improvement.

Table 2 summarizes the study phases, data sources, indicators, and analytical approaches used to assess each outcome.

**Table 2:** Operationalisation of study outcomes.

Implementation outcome	Study phase(s)	Data source(s)	Indicators	Analysis
Feasibility	Phases 1, 2a, 2b, 2c	Screening logs, PREM completion records, field notes, staff focus groups	Participation rate, completion rate, assistance required, barriers and facilitators to implementation	Descriptive statistics; qualitative content analysis
Acceptability	Phases 1, 2a, 2b, 2c	PREM completion records, field notes, participant feedback, staff focus groups	Willingness to participate, participant feedback, reported distress, perceptions of the measure and administration process	Descriptive statistics; qualitative content analysis
Appropriateness	Phase 2c	Staff focus groups	Perceived relevance, fit and usefulness of consideRATE for patients receiving specialist palliative care and the hospital context	Qualitative content analysis
Exploratory outcome	Study phase(s)	Data source(s)	Indicators	Analysis
Perceived value for quality improvement	Phase 2c	Staff focus groups informed by aggregated PREM data (including response distributions, top-box scores and free-text feedback)	Perceived usefulness of PREM findings for reporting, benchmarking, identifying improvement opportunities and supporting change	Qualitative content analysis

### Recruitment

#### Phase 1

The palliative care research nurse liaised with the nurse in charge of the inpatient specialist palliative care unit to identify eligible patients, who were then provided with study information and invited to complete the PREM. Only patients who met the eligibility criteria and were considered appropriate for the approach were included as eligible participation occasions within screening logs and participation-rate calculations. Participation was voluntary, and no patient identification details were recorded in the survey, with reassurance that involvement would not affect their immediate care. Completion of the PREM was taken as implied consent. Where patients were unable to complete the PREM, carers were invited to participate as their proxies.

#### Phase 2

##### Patients (phases 2a and 2b)

The research nurse worked with the nurse in charge of the specialist inpatient unit (2a) and the clinical nurse consultant supporting the hospital consultative service (2b) to identify patients who met study eligibility criteria. Clinical teams assisted in identifying patients considered appropriate for approach. Only patients meeting eligibility criteria and considered appropriate for approach were included as eligible participation occasions within screening logs and participation-rate calculations. Eligible patients were provided with study information and invited to participate.

Staff (Phase 2c) Recruitment for the Phase 2c focus groups occurred via email from the research team to eligible staff, inviting them to participate and providing the study information sheet and consent form. The researchers offered the opportunity to provide additional information as required. Interested staff were allocated to attend one of three focus group sessions depending on availability, and their written consent was obtained.

##### Patient-reported experience measure (PREM)

consideRATE (28) was chosen as the PREM to study due to early pilot work establishing its feasibility and acceptability within a different Australian hospital context (8). consideRATE is a brief, validated PREM designed to measure perceived quality of care for patients with serious illness (28, 32, 33). Developed using a user-centered design process involving patients, family members, and clinicians, consideRATE was designed to assess domains of care considered important to people living with serious illness. Initial psychometric testing found that the measure demonstrated acceptable content validity and construct validity, with subsequent studies supporting its usability and measurement performance in serious illness populations (28, 32, 33). consideRATE's eight questions are listed in Box 1.

BOX 1 **consideRATE's (28, 32) eight questions.**

**Table:** 

1.	**How would you rate our attention to your physical problems?** *things like pain, dry mouth, or trouble breathing*
2.	**How would you rate our attention to your feelings?** *things like feeling sad, worried, or like a burden*
3.	**How would you rate our attention to your surroundings?** *things like noise, light, or warmth*
4.	**How would you rate our respect for what matters to you?** *things like values, preferences about care, or important activities*
5.	**How would you rate our communication about your plans?** *things like medicines, procedures, or place of care*
6.	**How would you rate our attention to your affairs?** *things like financial planning and preparing enduring documents*
7.	**How would you rate our attention to what you can expect?** *things like illness getting worse or time left to live*
8.	**Are there any other things you want to share?**

The instructions provided for patients and carers refer to this PREM as being for “people who are ill or their carers” and as being about “the care you had from our hospital in the last few weeks” (28). Response options included “very good,” “good,” “bad,” “very bad,” and “doesn’t apply.” We modified consideRATE (28) by adding free-text response options, with approval from the original consideRATE authors (8, 9, 34). No changes to consideRATE question wording, question prompts, and measurement scale were made.

### Data collection

*Phases 1 and 2a, b—PREM collection:* The research nurse approached each eligible patient with a paper version of consideRATE and helped complete it as needed. Assistance included reading questions aloud, clarifying wording, and recording participants' responses. Screening log information and case report forms were completed to capture any feedback or questions about completing consideRATE, as well as completion details (e.g., who completed, assistance required, feedback). All paper-recorded data were entered into REDCap (35, 36) hosted by the study site for secure data management. Demographic details of participants were not captured given we were testing anonymous data collection and feedback.

PREM responses were collected, and no participant identifiers were retained. For participation analyses, eligible participation occasions were defined as occasions when patients met study eligibility criteria and were considered appropriate for approach by the clinical team. As repeat completion was permitted for patients with prolonged admissions (maximum once weekly), the unit of analysis for participation and completion reporting represents eligible participation occasions and PREM completion occasions rather than unique individuals. Consequently, the number of participants who completed consideRATE more than once could not be determined retrospectively.

*Phase 2—Staff focus group/s (Phase 2c):* Three focus groups were conducted in November and December 2025, each of 1-h duration. Focus group/s were facilitated by CV (lead researcher) via Teams, audio-recorded and transcribed using Microsoft Teams. A predetermined question route was used to guide discussion (Box 2), supported by a PowerPoint presentation and a data pack to provide context for the study. The data pack (example in Supplementary File 1) provided a distribution of responses for each consideRATE item summarized as frequencies and proportions (stacked bar charts) to identify patterns in reported care experience. Free-text responses were categorized into positive feedback or constructive feedback for ease of review.

BOX 2 **Focus group (staff) interview question route.**

**Table:** 

What do participants think about consideRATE as a PREM for patients with palliative care needs? Do participants like the way we have reported the data (stacked bar chart and comments in positive/constructive)? Would different formats be preferable? What do participants think about the collection of PREMs from patients with palliative care needs? Is this important in addition to the current Net Promoter Score (NPS)* data (or not), and why? How could you see these data being collected from your perspective? Do you feel these data would be helpful to inform improvement work? If so—what supports may be needed to inform and guide this work? Who do you think should be engaged in shaping how feedback like this is interpreted and used to inform improvements? OR What would help ensure any PREM used for palliative care patients is meaningful across different settings (acute & subacute)? Any other comments?

*The Net Promoter Score (NPS) (37) is a widely used measure of patient experience and loyalty that assesses how likely patients are to recommend a healthcare service to others, calculated as the percentage of promoters minus the percentage of detractors.

### Data analysis

*PREM data:* Data were separated into an inpatient cohort comprising completed PREMs from Phases 1 and 2a, and a consultative cohort comprising completed PREMs from Phase 2b. Analysis of PREM responses had two purposes: (1) to describe the pattern and completeness of data collected through consideRATE, and (2) to generate summary reports that were used during staff focus groups to explore the perceived usefulness of the data for quality improvement activities. Descriptive statistics were used to characterize feasibility and acceptability data, whereby participation and completion rates were calculated and compared against predefined feasibility thresholds (>70% participation and >80% full completion). Additional indices included the mode of completion (patient, carer, or assisted by the research nurse), and the proportion of these participants requiring this assistance. Because repeat completions could not be reliably identified within the de-identified dataset, all completed PREMs were included in descriptive analyses and in the aggregated data summaries provided to focus-group participants. PREM response distributions were examined descriptively. Consistent with our prespecified analysis plan and informed by previous studies using consideRATE that identified substantial positive skew in response distributions (33, 34), top-box scoring (the proportion of “very good” responses) was calculated alongside descriptive summaries to enhance discrimination between domains and support identification of potential areas for improvement. These summaries were incorporated into the reports provided to focus-group participants.

*Staff focus groups (Phase 2c):* Audio-recorded focus group data were transcribed (via Teams) and analyzed using qualitative content analysis informed by the study's implementation outcomes (feasibility, acceptability, appropriateness, and perceived value for quality improvement). Coding was primarily deductive, using these implementation domains as an organizing framework, while allowing inductive identification of subcategories and explanatory patterns within each domain. Initial coding was undertaken by the lead researcher and was iteratively refined through discussions with MR and DS, who reviewed coding decisions, category development, and interpretation of findings. Differences in interpretation were resolved through discussion and consensus. The focus group question route was informed by implementation science concepts, including the Theoretical Framework of Acceptability, and explored participant perspectives regarding the acceptability, appropriateness, feasibility, and practical implementation of consideRATE. Focus groups were facilitated by CV, an experienced palliative care researcher with a longstanding interest in patient experience measurement and quality improvement. Recognizing that researchers' backgrounds and perspectives may influence interpretation, the wider multidisciplinary research team was involved to challenge assumptions and enhance analytical rigor. Findings are presented by major implementation topics explored during the focus groups to support practice-oriented recommendations.

### Ethical consideration

This study was reviewed by the St Vincent's Hospital Human Research Ethics Committee and approved on 4 April 2024 (2023/ETH00591). Given the clinical vulnerability of participants, including people with advanced illness and their carers, the study was classified as greater than low risk. Several strategies were implemented to minimize risk to patient and carer participants. Participation was voluntary, and it was clearly communicated that involvement would not affect clinical care. Study processes were conducted independently of the treating clinical team at the individual participant level to reduce perceived coercion. The consideRATE measure was selected due to its brevity and prior evidence of acceptability in similar populations. The research nurses collecting data from patients and carers had expertise in engaging people in palliative care research and were trained to identify and respond to signs of fatigue or psychological distress. Participants were able to pause, defer, or withdraw from participation at any time without consequence. If distress was identified, the research nurse responded supportively and, when appropriate and with permission, liaised with the treating clinical team to ensure follow-up care.

## Results

### Feasibility and acceptability of completing the consideRATE questions

Across 219 eligible participation occasions (defined as patients meeting eligibility criteria and considered appropriate for approach), 188 PREMs were completed (86% participation rate), for which assistance was provided by the research nurse for 94% (Table 3).

**Table 3:** consideRATE participation and completion rates by study phase (predefined feasibility thresholds were >70% participation and >80% full completion).

Outcome	Phase 1	Phase 2		Overall (*N* = 219)
	Inpatient unit (*n* = 10)	Inpatient unit (*n* = 74)	Consultative service (*n* = 135)	
Agreed to participate, n/eligible occasions (%)	9 (90%)	53 (72%)	127 (94%)	189 (86%)
Fully completed PREM, n/consenting occasions (%)	8 (89%) (5 patients, 3 carers)^a^	53 (100%) (48 patients, 5 carers)^a^	127 (100%) (122 patients, 5 carers)^a^	188 (99%) (175 patients, 13 carers)^a^
Received assistance during attempted PREM completion, n/consenting occasions (%) (all patient participants)	5/9^b^ (56%)	51/53^b^ (96%)	121/127^b^ (95%)	177/189^b^ (94%)

a Participants reflect occasions of completing the PREM rather than individual participants.

b Assistance denominators are based on all consenting participants because assistance was offered during attempted PREM completion, irrespective of whether the PREM was subsequently completed.

#### Phase 1—inpatient palliative care unit

Ten eligible inpatients were identified during Phase 1, of whom nine consented to participate. Of those who consented, eight completed the PREM, including five patients and three carers. Assistance from the research nurse was required for five of the eight participants who completed the measure. Despite meeting the prespecified participation and completion rates, eligibility based on being ready for discharge home meant only a small proportion of the overall palliative care unit population could be involved, and therefore, this approach was not deemed feasible in the long term.

#### Phase 2a—inpatient palliative care unit

Seventy-four eligible inpatients were approached, of whom 53 consented to participate (72%). Twenty-one patients declined participation due to fatigue (*n* = 13, 62%), breathlessness (*n* = 3, 14%), or prior consideRATE completion (*n* = 5, 24%). Of those who agreed to complete the consideRATE questions, all 48 patient occasions and 5 carer occasions were completed in full (100%), with 96% requiring assistance from the research nurse.

#### Phase 2b—consultative palliative care service (public hospital)

A total of 135 eligible inpatients were approached, of whom 127 consented to participate (94%). Eight patients declined participation due to fatigue (*n* = 5, 63%) or because they had completed consideRATE the week prior and did not wish to do so again (*n* = 3, 37%). Of those who agreed to complete consideRATE, all (122 patient occasions and 5 carer occasions) completed all questions (100%).

Across the phases, supported administration was common, with assistance provided during 56% of consenting occasions in Phase 1, 96% in Phase 2a, and 95% in Phase 2b. Assistance was generally provided by the research nurse and included reading questions aloud, clarifying wording, and recording participants' verbal responses. The reasons assistance was required were not systematically coded. Proxy completion by carers occurred on 13 occasions. Regarding acceptability, no patients or carers expressed distress about being approached to participate or while completing consideRATE. Field notes documented occasional spontaneous feedback from participants, including two patients who expressed appreciation for the opportunity to provide feedback about their care.

### Responses to the consideRATE questions

Across both inpatient (Phases 1 and 2a; *n* = 61 completed PREMs) and consultative cohorts (Phase 2b; *n* = 127 completed PREMs), item-level completion was 100%, with no missing data across all consideRATE domains. In contrast to other consideRATE questions, a large proportion (42%) of the response “doesn't apply” was noted for question 6, which focuses on attention to affairs, such as a will, finances or enduring documents for care. Across all domains, responses demonstrated a marked positive skew, with most ratings falling within the “very good” and “good” categories in both the inpatient and consultative groups (Supplementary File 1). Negative ratings (“bad” or “very bad”) were infrequent but present across several domains, particularly in relation to communication about plans and the surroundings/environment.

Analysis of field notes recorded by the research nurse while collecting consideRATE ratings found that barriers to participation were primarily related to patient condition (*n* = 21 occasions) and cognitive burden (*n* = 2 occasions). Fatigue was the most common reason for declining participation (*n* = 18 occasions). Two participants who completed consideRATE noted difficulty interpreting or applying the questions to their individual circumstances due to prolonged admissions or complex care trajectories.

### Staff perspectives on the appropriateness, implementation and usefulness of consideRATE

There were 15 participants overall (6 male, 9 female), who included Nursing Unit Managers (*n* = 7) across a range of specialties (surgical, aged care, hematology/bone marrow transplant, and inpatient palliative care), palliative care clinicians (consultants *n* = 2; CNC *n* = 1), allied health (social worker *n* = 1), service leadership roles (service manager *n* = 1; end-of-life care coordinator *n* = 1), and quality, safety, and data personnel (*n* = 2).

Findings are presented in line with the focus group question route.

1. Perceived value of consideRATE as a PREM

Participants expressed support for consideRATE, describing it as a meaningful and relevant measure of patient experience for people with palliative care needs. They thought the tool provided more nuanced and actionable insights than existing PREMs such as the Net Promoter Score (NPS) (37) or the Australian Hospital Patient Experience Question Set (AHPEQS) (4). Staff participants reported the data as relevant, meaningful, and directly applicable to informing care delivery and improvement. They identified that a key strength was the palliative care focus of the questions, which enabled exploration of domains not captured elsewhere, particularly end-of-life communication and holistic care:

It does provide that extra level of information that we wouldn't necessarily ask… it's got those really specific questions around palliative care.

Participants also emphasized the importance of capturing the patient voice directly, noting the high proportion of responses completed by patients rather than proxies as a key strength:

The majority of responses are from patients… that's the important part of what we're doing.

2. Interpretation and usefulness of PREM data for improvement

Many participants described the data as highly actionable, enabling identification of priority areas for improvement at both the ward and system levels. The ability to identify specific domains (e.g., communication and environment) and link these to improvement strategies was highlighted:

It gives them [multidisciplinary colleagues] an idea on what things we need to focus on… especially with the communication part… that's something that we could then work on.

Free-text responses were particularly valued for their granularity and explanatory power, although participants emphasized the need for them to be grouped into themes:

I'm interested in the constructive feedback… group them together with similar themes rather than each individual quote.

Participants also noted that PREM data could support advocacy for change, particularly for system-level issues:

It helps to advocate for certain things to be done.

3. Preferences for data reporting and interpretation

Participants identified the need for dual-layered reporting to meet the needs of different audiences. Detailed data (e.g., stacked bar charts and themed qualitative feedback, Supplementary file 1) for clinical teams were seen as useful for guiding practice-level improvement:

For me it's a clear visualisation… easy to quickly scan and see where you need to focus.

However, participants emphasized that executive staff require simplified metrics, particularly a single performance indicator based on top-box (“very good”) scoring:

From an executive point of view… I want to know what the “very good” is and whether we're meeting target.

There was also strong support for benchmarking (e.g., ≥ 80%), trend analysis over time, and integration into existing reporting systems [e.g., Microsoft Power BI (Business Intelligence) dashboards]. Participants identified top-box reporting as a useful metric for executive reporting, benchmarking, and quality-improvement activities.

4. Perceived importance of PREMs for patients with palliative care needs

Participants agreed that PREM data for patients with palliative care needs are both important and currently lacking. The data were seen as addressing a critical gap in existing patient experience systems:

It's a patient group that has been excluded from PREMs… and it's such important work to collect it.

Importantly, participants emphasized that the data reflect whole-of-system care rather than only specialist palliative care input:

It's not about palliative care services… it's about whether the needs… were met or not met.

5. Feasibility and logistics of PREM data collection

While participants supported PREM collection in principle, they consistently highlighted significant challenges related to implementation, particularly workforce constraints. Participants commonly expressed concern about integrating PREM collection into routine nursing workloads:

It's too much to ask the nurses… there's just no scope there.

Participants suggested the use of dedicated staff or research personnel to collect PREM data from patients with palliative care needs, or the potential use of trained volunteers for this purpose, and/or focusing on time-limited “snapshot” data-collection models.

6. Requirements for implementing data-informed improvement

Participants described the need for structured, multidisciplinary approaches to data interpretation and improvement, with a strong emphasis on local ownership supported by organizational systems. Improvement work was seen as most effective when led at the ward level and involving multidisciplinary teams:

Each ward would need to bring together their own team… nursing, social work, medical reps…

However, participants also emphasized the importance of executive engagement, particularly for system-level issues such as the environment and infrastructure:

A number of the things… local teams don't have influence over… executives need to be engaged.

Existing hospital structures (e.g., quality improvement and data teams) were identified as key enablers of implementation and sustainability.

## Discussion

This two-phase prospective pilot study in a specialist palliative care unit and consultative service demonstrated consideRATE participation and completion rates that met or exceeded predefined feasibility thresholds, with most patients and carers supported by research staff to complete the PREM and none reporting that it caused distress. Staff participants considered consideRATE appropriate and perceived the resulting data to be meaningful and actionable for quality improvement purposes (exceeding the perceived utility of existing PREMs). Although participation within the inpatient unit cohort was only slightly above the prespecified threshold, the findings suggest that patients wished to provide feedback on their care experiences and that the tool was manageable even among a clinically complex, seriously ill population.

These findings build on our earlier evaluation of consideRATE within Australian acute hospital wards (8, 34). Similar to previous work, participation rates among patients and carers were high, and staff valued the specificity and actionability of the feedback, particularly when supported by free-text comments. However, this study extends previous knowledge in several important ways. First, it demonstrates that supported administration is likely to be essential in inpatient specialist palliative care settings, with almost all patient participants requiring assistance to complete the consideRATE questions. Second, it identifies a key implementation challenge: despite strong staff support for collecting PREM data, routine and sustainable implementation is unlikely to be feasible without dedicated personnel, alternative workforce models, and integration into existing organizational quality systems. Finally, the replication of a high proportion of “does not apply” responses to the item relating to financial and legal affairs suggests that further qualitative exploration of this question is warranted within Australian palliative care contexts (8, 34).

The manner in which consideRATE was administered proved to be critical to its successful use. A structured screening process ensured that feedback was collected from the population for whom the measure was designed, namely patients living with advanced illness (32). Nearly all participants required assistance to complete the measure, highlighting the importance of interviewer-supported administration in specialist palliative care. The research nurse played a central role by reading questions aloud, clarifying wording, and recording responses. Although PREMs are commonly administered through self-completed, telephone, or interviewer-administered approaches (38, 39), our findings suggest that unsupported administration would exclude many patients because of fatigue, functional limitations, and the cognitive burden associated with serious illness. Supported administration therefore appears necessary to ensure equitable participation in patient experience measurement among this population. While this approach facilitated high participation and complete datasets, its resource requirements raise important questions regarding scalability, as staff consistently identified workforce capacity as a major barrier and regarded dedicated personnel as essential for routine implementation.

Higher participation among patients receiving consultative palliative care, compared to the palliative care inpatient unit where there were more refusals due to fatigue and breathlessness, also offers useful implementation insights. Nevertheless, overall participation and completion met predefined targets, demonstrating that many patients receiving specialist palliative care can provide meaningful feedback with appropriate support.

Findings also highlight the importance of tailoring data presentation to different stakeholders. Clinical staff valued detailed, domain-specific feedback to guide improvement, whereas executive staff were said to prefer summarized metrics and benchmarking. This supports the need for dual-level reporting embedded within existing organizational dashboards and quality systems. More broadly, the value of PREMs lies not simply in collecting patient experience data, but in using those data to drive improvement (34). Evidence suggests that achieving and sustaining change in acute hospital settings requires implementation frameworks that support ongoing review, interpretation, and action (34, 40–43). Given that approximately a third of hospital inpatients have palliative care needs (13, 14), establishing systematic approaches to understanding and responding to care experiences within this population represents an important priority for quality improvement efforts (34, 40). Although staff supported this PREM in principle, they emphasized that meaningful and sustained change will require dedicated resourcing and organizational commitment to support teams to engage with, interpret, and act on PREM findings (44).

### Recommendations for future practice

1. Consider establishing a resourced, sustainable model for PREM data collection using consideRATE for patients with palliative care needs.
2. Identify and test organizational processes to support thematic analysis, data interpretation and feedback of consideRATE data.
3. Subject to 1 and 2, integrate consideRATE into routine organizational quality systems and reporting pathways to inform quality improvement.

### Recommendations for future research

1. Explore the process, outcomes, and required supports for other groups of staff (e.g., volunteers) collecting PREM data using consideRATE for patients receiving specialist palliative care.
2. Explore factors contributing to high rates of “not applicable” responses for consideRATE question 6.
3. Examine how PREMs may capture both the specific contribution of specialist palliative care services and broader hospital care, particularly within consultative models of palliative care where clinical responsibility is shared across multiple teams.
4. Examine whether PREM responses differ according to mode of completion (self-completed, interviewer-assisted, or proxy-completed) and explore the potential influence of supported administration on response patterns and interpretation of questions.

### Strengths and limitations

This study has several strengths. It represents one of the few prospective, real-world evaluations of PREM implementation among patients receiving inpatient specialist palliative care, directly addressing a recognized gap in patient experience measurement for this population. The inclusion of both inpatient and consultative palliative care cohorts across two study phases strengthens the applicability of findings across different models of hospital care, while the participation and complete item-level response rates support the robustness of the feasibility conclusions. The use of multiple data sources, including screening logs, field notes, free-text responses, and multidisciplinary clinician focus groups, enabled examination of patient- and system-level feasibility and acceptability. Importantly, the study captured patients' perspectives rather than relying predominantly on proxy or post-discharge reporting, a notable strength given the historical exclusion of palliative care recipients from PREM initiatives.

Several limitations should be considered. As a single-site study conducted within a well-resourced metropolitan health service, the findings may not be directly transferable to settings with different patient populations, service structures, or resource availability. There is the potential for selection bias arising from both the recruitment process and participant self-selection. Although clinical teams assisted with identifying patients appropriate for the approach to minimize participant burden and ensure ethical recruitment, this process may have resulted in under-representation of patients with greater symptom burden, functional or cognitive impairment, or clinical instability. Consistent with this possibility, fatigue and breathlessness were common reasons why patients declined to participate. Consequently, participation, completion, and feasibility estimates may reflect the experiences of patients who were relatively well and may not fully represent all patients receiving specialist palliative care. The absence of demographic and clinical data also limited the assessment of representativeness and prevented the exploration of potential differences between participants and non-participants or across patient subgroups. In addition, not collecting patient identifiers prevented us from identifying repeat respondents and limited our ability to distinguish unique participants from repeat completion occasions, which may have influenced participation and completion estimates. Although targeting patients receiving specialist palliative care was a strength, findings may not be generalizable to all inpatients with palliative care needs. Consultative patients received care from both specialist palliative care clinicians and ward-based treating teams, meaning that PREM responses may have reflected experiences of the broader hospital system rather than specialist palliative care alone.

Acceptability was not assessed using a structured acceptability instrument or a standardized post-completion evaluation. Instead, conclusions regarding acceptability were based on participation and completion rates, the absence of observed or reported distress, spontaneous participant feedback, and clinician perspectives. Consequently, dimensions of acceptability such as burden, satisfaction, perceived relevance, and ease of completion were not systematically measured and warrant further investigation.

A further limitation relates to the supported administration model used throughout this study. While research nurse assistance enabled participation by people who may otherwise have been excluded due to fatigue, functional limitations, or illness burden, this involvement may have influenced how some participants interpreted questions or responded. Research nurse involvement also limits scalability in settings where this staff resource is less readily available. Both patient and proxy responses were included in the analyses, and their respective perspectives may not be directly comparable, nor was the study designed to examine differences among patient, proxy, self-administered, and interviewer-assisted responses.

Finally, focus group participants were predominantly drawn from leadership, specialist palliative care, and quality-improvement roles. Consequently, findings may reflect the perspectives of staff with particular interest or expertise in service improvement and may not fully represent the views of frontline clinicians responsible for routine PREM administration.

## Conclusions

Supported administration of consideRATE in a specialist palliative care unit and consultative service resulted in high participation and completion rates with no reported distress among participating patients and carers. Staff considered the measure appropriate and the data useful for informing quality improvement, but sustainable routine implementation was considered likely to require dedicated human resources and integration within the hospital's quality systems. Further research is needed to identify scalable and resource-efficient approaches to PREM collection for inpatients with palliative care needs.

## Data Availability

The original contributions presented in the study are included in the article/Supplementary Material, further inquiries can be directed to the corresponding author.
