# Supplementary material for: The utility of the “consideRATE” patient-reported experience measure in hospital patients receiving specialist palliative care: a two-phase prospective pilot study
Source: Front Health Serv. 2026 Sep 30;6:1933654. doi: 10.3389/frhs.2026.1933654 (PMC13662150; doi:10.3389/frhs.2026.1933654)
Supplement: Supplementary file 1 [file Supplementaryfile1.docx]

**Supplementary file 1**

**Example of data presentation for the focus groups – Phase 2c**

**Table 1 – Phase 2a rating data from consideRATE (PREM) – inpatient palliative care unit**

**Table 2 - Phase 2b rating data from consideRATE (PREM) – inpatients in a public hospital referred to specialist palliative care**

Free‑text responses provided per question, gathered important contextual insight into patient experience. Comments were categorised as positive or constructive, with themes consistently identified across both datasets (inpatient and public hospital patients known to specialist palliative care). Positive feedback overwhelmingly emphasised the relational aspects of care, including staff compassion, attentiveness, and the ability to feel heard and supported.

The care team’s sensitivity and compassion to my feelings and burdens are exceptional (patient – consult service)

Patients also highlighted experiences of holistic and person‑centred care, including attention to emotional needs, family involvement, and spiritual support. A strong sense of safety, trust, and confidence in care was evident across responses.

In all aspects they have respected my perception of my values with respect and dignity. Everyone has been sensitive, caring and compassionate (patient – consult service)

Constructive feedback clustered around several consistent domains. Communication emerged as the most prominent area for improvement, with participants describing gaps in coordination between clinical teams, limited updates, and unclear care plans.

Confused with departments that offer different things. It seems that they are not communicating with each other. For example, radiology rang about an appointment, but I needed wound care (patient – consult service)

Environmental factors, including noise, temperature, and sleep disruption, were also frequently reported.

There are so many noises and people are snoring. There are constant environment noises and nurses doing obs in the middle of the night. Last night the nurse was good and did my obs at 10 pm so I could sleep (patient – consult service)

Issues related to food quality and choice were consistently identified, alongside broader system-level concerns such as delays, fragmented processes, and perceived staffing limitations.

Food is terrible, the hot meals are really bad. Need to pay more attention (patient – consult service)

A smaller but important set of responses highlighted unmet psychosocial needs, including limited attention to emotional concerns in some cases.

Often not sympathetic to my needs. They need to respect me and my condition. (patient – consult service)
